# Winning the game: brain processes in expert, young elite and amateur table tennis players

**DOI:** 10.3389/fnbeh.2014.00370

**Published:** 2014-10-27

**Authors:** Sebastian Wolf, Ellen Brölz, David Scholz, Ander Ramos-Murguialday, Philipp M. Keune, Martin Hautzinger, Niels Birbaumer, Ute Strehl

**Affiliations:** ^1^Faculty of Science, Institute of Clinical Psychology and Psychotherapy, University of TuebingenTuebingen, Germany; ^2^Department of Internal Medicine VI: Psychosomatic Medicine, University Hospital TuebingenTuebingen, Germany; ^3^Institute of Medical Psychology and Behavioral Neurobiology, University of TuebingenTuebingen, Germany; ^4^TECNALIA, Health-TechnologiesSan Sebastian, Spain; ^5^Department of Neurology, Klinikum BayreuthBayreuth, Germany; ^6^Department of Physiological Psychology, Otto-Friedrich-UniversityBamberg, Germany; ^7^Ospedale San Camillo, Istituto di Ricovero e Cura a Carattere Scientifico (IRCCS)Venice, Italy; ^8^German Center for Diabetes ResearchTuebingen, Germany

**Keywords:** ERD/ERS, EEG, sensorimotor rhythm, elite athletes, table tennis, motor efficiency, motor skill

## Abstract

This study tested two hypotheses: (1) compared with amateurs and young elite, expert table tennis players are characterized by enhanced cortical activation in the motor and fronto-parietal cortex during motor imagery in response to table tennis videos; (2) in elite athletes, world rank points are associated with stronger cortical activation. To this aim, electroencephalographic data were recorded in 14 expert, 15 amateur and 15 young elite right-handed table tennis players. All subjects watched videos of a serve and imagined themselves responding with a specific table tennis stroke. With reference to a baseline period, power decrease/increase of the sensorimotor rhythm (SMR) during the pretask- and task period indexed the cortical activation/deactivation (event-related desynchronization/synchronization, ERD/ERS). Regarding hypothesis (1), 8–10 Hz SMR ERD was stronger in elite athletes than in amateurs with an intermediate ERD in young elite athletes in the motor cortex. Regarding hypothesis (2), there was no correlation between ERD/ERS in the motor cortex and world rank points in elite experts, but a weaker ERD in the fronto-parietal cortex was associated with higher world rank points. These results suggest that motor skill in table tennis is associated with focused excitability of the motor cortex during reaction, movement planning and execution with high attentional demands. Among elite experts, less activation of the fronto-parietal attention network may be necessary to become a world champion.

## Introduction

Motor efficiency plays a crucial role in determining athletic skill, characterized by automaticity, speed and accuracy. Experts in a highly reactive sport, such as table tennis, move rapidly, effortlessly and smoothly. Motor efficiency can be achieved through intensive training, which leads to improved perception, focus, anticipation, planning and fast responses (Yarrow et al., 2009). These skills are especially important in a fast pace sport like table tennis, where athletes have to process many cues simultaneously in order to react appropriately. Studies of self-paced movements using electroencephalography (EEG) found a frequency increase and a desynchronization with a reduction in amplitude in the 8–15 Hz frequency band recorded from sensorimotor areas (sensorimotor rhythm, SMR). A desynchronization of the SMR prior to and during a self-paced movement is called movement event-related desynchronization (ERD) and is linked to activation of the central motor system. An increase in SMR is referred to as event-related synchronization (ERS) and is associated with inhibition of the motor cortex (Pfurtscheller et al., 1996; Pfurtscheller and Lopes Da Silva, 1999; Klimesch et al., 2007, 2011). Synchronization in the SMR rhythm (ERS) is often observed at electrodes recording from task irrelevant brain areas during cognitive (Worden et al., 2000; Sauseng et al., 2005) or motor tasks (Alegre et al., 2004): For example, after movement onset, the contralateral motor cortex desynchronizes while the ipsilateral cortex and the surrounding areas synchronize. This pattern has been named focal ERD surrounded by ERS (Pfurtscheller and Neuper, 1994, 2006). However, recent findings in healthy participants (Ramos-Murguialday and Birbaumer, under review) and in stroke patients (Antelis et al., 2012) observed ERD in contralateral and ipsilateral electrodes. ERD can be divided into two functionally and topographically different components: 8–10 Hz ERD and 10–12 Hz ERD. The low-frequency ERD occurs especially when movements become more complex and is widespread over the entire scalp in all cortical areas involved in a motor task including primary sensorimotor, premotor and parietal areas. 8–10 Hz ERD seems to be further related to attentional processes during motor tasks (Klimesch, 1999; Pfurtscheller and Lopes Da Silva, 1999; Pfurtscheller et al., 2000; Klimesch et al., 2007). The high-frequency ERD is restricted to sensorimotor areas and seems to reflect processing specific to simple motor-tasks (Klimesch, 1999; Pfurtscheller and Lopes Da Silva, 1999; Pfurtscheller et al., 2000; Klimesch et al., 2007).

In the context of athletic performance several authors stated that elite athletes require less brain activation (weaker ERD) in task relevant brain areas compared to novices, which had led to the formulation of the “neural efficiency” hypothesis in the athletic context (Babiloni et al., 2010). In line with these assumptions, the EEG literature emphasizes that low-frequency and high-frequency ERD is weaker in elite athletes. Babiloni et al. (2009) for example found that elite rhythmic gymnasts showed weaker low- and high frequency alpha ERD compared to non-gymnasts in occipital and temporal areas and in the dorsal pathway while observing rhythmic gymnastics videos. A similar study found, that low- and high-frequency alpha ERD was weaker in dorsal and fronto-parietal pathways in elite karate athletes compared to amateurs and non-athletes while watching and judging karate videos (Babiloni et al., 2010). Karate and fencing athletes showed weaker low-frequency alpha ERD at left central, right central, mid-parietal, and right parietal areas and weaker high-frequency alpha ERD at right frontal, left central, right central, and mid-parietal areas during a monopodalic upright standing task compared to non-athletes (Del Percio et al., 2009b). Elite pistol shooters showed weaker low- and high-frequency alpha ERD than non-athletes over the whole scalp in a shooting task and best shots were correlated with right parietal and left central high-frequency ERS (Del Percio et al., 2009a). Furthermore, compared to amateur rifle shooters, elite shooters showed stronger occipital alpha ERS during a pre-shot period and stronger high-frequency alpha ERS over left central electrodes (Haufler et al., 2000). In sum, these EEG studies indicate that skilled athletes show less cortical activation (weaker low- and high frequency ERD) when performing tasks specific to their discipline.

However, some EEG literature emphasizes, that athletic expertise is associated with increased brain activation in task relevant brain areas (stronger ERD). High-frequency alpha ERD was stronger in successful compared to unsuccessful putts over the frontal midline and the arm and hand region of the right primary sensorimotor area in expert golfers (Babiloni et al., 2008). Research on motor related ERD in the athletes has mainly focused on self-paced sports like shooting, golf or gymnastics. However, highly reactive sports are different from self-paced sports: They require the perception and integration of quickly changing visual, auditory and tactile cues. Thus highly reactive sports are similar to complex procedural multisensory integration motor tasks. There is evidence indicating that these tasks produce increased brain activation in premotor, motor and visual areas with the development of expertise or automaticity (Weisberg et al., 2007; Waldschmidt and Ashby, 2011). Karni et al. (1995) used fMRI to show stronger activity in the primary motor cortex after learning a complex motor task. An EEG study with karate and fencing athletes during an upright standing task, which required the integration of visual cues for body sway indicated stronger high-frequency ERD at right ventral centro-parietal electrodes in elite compared to novice athletes (Del Percio et al., 2007). Overall, evidence whether expertise in sports is correlated with reduced or increased cortical activation in task relevant brain areas is still ambiguous, indicating a need for larger and controlled studies to clarify this issue.

We are not aware of any published study examining athlete’s cortical activation patterns in table tennis athletes and suggest, that demands of table tennis and complex motor learning tasks are highly similar. Therefore we expect stronger activation in motor and fronto-parietal areas in expert table tennis players. We further assume, that attentional processes play a more important role in highly reactive sports than in self-paced sports and that movements in table tennis are much more complex. We hypothesize that table tennis athletes show predominantly ERD in the low-frequency alpha band (8–10 Hz), that seem to reflect general motor attentional processes and occurs predominantly in complex motor tasks (Klimesch, 1999; Pfurtscheller et al., 2000; Klimesch et al., 2007).

Most EEG studies looking at athletes have focused on differences between experts and novices, who are entirely unfamiliar with the specific athletic discipline. Thus, it is still unclear if the observed differences in cortical activation represent a psychophysiological mechanism induced by the amount of training that underlies superior performance in sports. To assure that the activation differences are dependent on expertise, we added young elite athletes as a group with an intermediate skill level and assumed that the cortical activation lies in between experts and amateurs. We developed a research design to compare different skill level groups from the same discipline and attempted to answer the following questions:

1. Do table tennis experts show higher activation of the motor and fronto-parietal cortex, reflected by more pronounced 8–10 Hz ERD, compared to amateurs in the end of a sport specific motor task in a competitive situation?
2. Does 8–10 Hz ERD in the motor cortex correlate positively with world rank points in expert table tennis players?

## Material and methods

### Participants

A total of 60 table tennis players were recruited from the German Table Tennis Association (DTTB, experts: *N* = 16), clubs from regional district leagues around Tuebingen, Germany (amateurs: *N* = 19) and from the squad of the Table Tennis Association of Baden-Wuerttemberg (TTBW), Germany (young elite athletes: *N* = 25). All young elite athletes were the best upcoming teen table tennis players in Baden-Württemberg with a realistic chance to become an elite expert in their career. Inclusion criteria were: right-handedness, 14–36 years of age, no medical disorders, no use of medication or drugs and no pregnancy. Of the recruited participants, 14 experts (5 female), 15 amateurs (4 female) and 15 young elites (6 female) met all criteria and were included in the analysis. There were no differences in the distribution of gender between groups ($c_{\left(2,44\right)}^{2}$ = 0.62, *p* = 0.73) and no difference in age between experts (*M* = 23.8, *sd* = 4.86) and amateurs (*M* = 22.8, *sd* = 4.16). Naturally, young elite athletes (*M* = 14.9, *sd* = 0.96) were younger than experts (*p* < 0.001) and amateurs (*p* < 0.001). Subjects signed an informed written consent and received monetary compensation for participation. All experiments reported here were approved by the local ethics committee and are in accordance with the Declaration of Helsinki.

### Experimental paradigm

We chose a motor imagery paradigm to minimize electromyographic (EMG) artifacts in the EEG data. Cortical activation patterns of real motor action and kinesthetic motor imagery, are similar (Porro et al., 1996; Roth et al., 1996; Jeannerod, 2001; Naito et al., 2002; Ehrsson et al., 2003; Neuper et al., 2005), although activation is stronger during active movements (Pfurtscheller and Neuper, 1997; Jeannerod and Frak, 1999). There is also evidence, that cortical activation during the preparatory period of motor imagery is similar to the activation preceding real movements (Kranczioch et al., 2009) although the preparation for real movements induces larger activations (Ramos-Murguialday and Birbaumer, under review). The mere observation of movements appears to activate the same sensorimotor areas that are involved in motor preparation and motor programming (Grezes and Costes, 1998; Babiloni et al., 2009, 2010; Halder et al., 2011). Based on these findings, we assume motor imagery to be a reliable and valid experimental paradigm to test our hypothesis. Participants were instructed to imagine themselves (kinesthetic motor imagery) playing table tennis, i.e., reacting to an opponent serving a ball presented in different video formats (for detailed information about the task please refer to paragraph 2.4 “task” and Figure 2).

**Figure 1:**
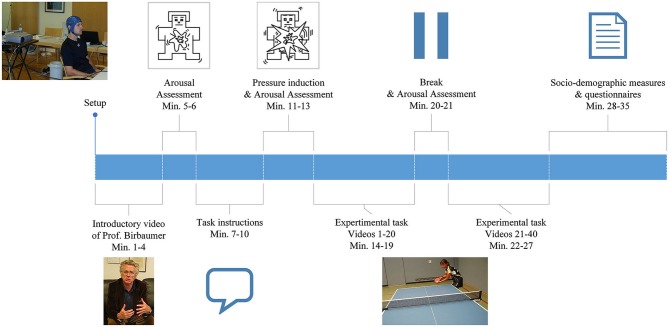
**Illustration of the general procedure as described in the Materials and Methods section**.

**Figure 2:**
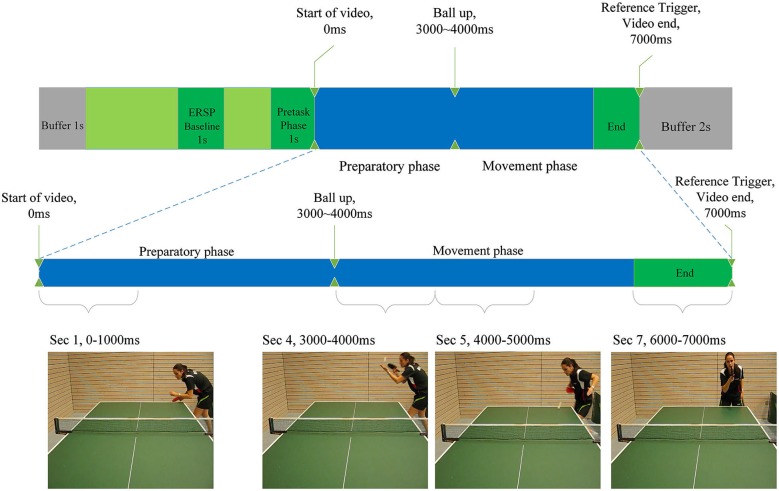
**Illustration of the experimental task as described in the Materials and Methods section**. Indicated are baseline period for the calculation of ERD/ERS, the start and end of the video clips and the differentiation between preparatory and movement phase. Screenshots of each phase are presented.

### Procedure

For an overview of the general procedure, please refer to Figure 1. Data was collected at a training center in Faak, Austria (male experts), at a training center in Oberhof, Germany (female experts), at the Institute of Medical Psychology, University in Tuebingen, Germany (amateurs) and at the Landessportschule in Tailfingen, Germany (young elite athletes).

EEG was recorded continuously while subjects sat on a chair facing the wall, on which all instructions and the table tennis videos were projected. At the beginning of the experiment subjects watched a video of Prof. Birbaumer, explaining the importance of the study in order to standardize the introduction and to induce motivation. Following the introduction, the player’s level of arousal was self-rated using the Self Assessment Mannequin (SAM; Bradley and Lang, 1994). Participants received detailed instructions projected onto the wall. They were then presented with a total of 40 video-clips of a table tennis player serving a ball. In each of the clips, they had to imagine the score to be 10:10 in the last set of an important match and react to the serve with a specific table tennis stroke: a forehand top spin. In order to further enhance ecological validity and to meet the degree of mental pressure experienced in a real competition situation, the athletes were told the imagined forehand topspins of all participants will be ranked in order of intensity and quality based on the EEG recording. Immediately after this pressure induction the level of arousal was self-rated again using the SAM questionnaire. There was a break (duration was chosen by the participant) after the first 20 videos and a 5 s break after each video. During the 5 s break the sentence “10:10 last set” was displayed and subjects were instructed to look at this sentence in order to prevent distraction and to ensure a competitive mindset. After the task, subjects were given questionnaires to assess demographic and control variables.

All experiments were carried out in a quiet room using the same EEG equipment, software and set up (beamer, notebooks, screen, distance from the participant to the screen). All instructions for the task were presented with E-Prime (Science Plus Group BV, Groningen, Netherlands). Start and end triggers of the videos were sent from E-Prime via a trigger interface (NeXus Trigger Interface, Mind Media, Herten, Holland) to a Lenovo Thinkpad T500 Notebook on which all EEG data were recorded.

### Task

Each video trial lasted 7 s (see Figure 2). Between the 3^rd^ and the 4^th^ second the player in the video throws up the ball (the ball leaves the passive hand of the opponent), to perform the serve. We termed the first 3 s of the video to comprise the “preparatory phase” in which the return movement is planned, because height, speed, rotation and direction of the arriving ball can only be anticipated when the opponent’s specific wrist-, arm- and shoulder movements, racket angle and body orientation can be perceived (Singer et al., 1996).

Seconds 4, 5 and 6 of each trial comprise the “movement phase”, during which we assume the subject imagines the execution of the forehand topspin. Four videos with different serves were presented 10 times in random order. Each serve was either directed more to the middle or to the right side of the table and was served with back- or sidespin. The player in the video served the ball in a way (length, height, rotation) to make a forehand top spin the most appropriate return option. This was confirmed by several expert trainers, who evaluated all videos and consistently regarded a forehand top spin as a suitable return. The forehand topspin was chosen, because it is one of the most important and profitable table tennis strokes.

To ensure ecological task validity and motivation of the participants, skill level and gender of the player in the video were adapted for each group. The videos of the young elite table tennis players were further adapted for younger (under 15 years of age) and older (under 18 years of age) participants. This resulted in 8 different video sets comprising each of four different videos in which the ball was presented to two directions (forehand side or middle side of the table) and with two different rotations (sidespin or underspin).

All videos were recorded in a coliseum, standardized in length, height of table, direction, rotation and sequence of the serve, the table set-up and were presented without sound. The only differences between videos were the table tennis player’s clothes, age, gender and handedness: only male experts watched a left-handed opponents. Female experts, all amateurs and all young elite athletes watched right-handed table tennis players.

### EEG

EEG was recorded from 21 electrodes using a 32-channel system with sintered ring-electrodes with carbon coating and active shielding (Nexus 32, Mind Media B.V., Herten, Holland) according to the 10–20 system (Jasper, 1958). A common average reference was used to minimize biases introduced by single electrode referencing (Qin et al., 2010). DC offset was kept below 25 µV during the whole recording. The signal was amplified with a NeXus 32 amplifier (Mind Media B.V., Herten, Holland) using a 24 bit A/D conversion, and was digitized at a rate of 2048 Hz. Data was exported in EDF+ format and imported to Brain Vision Analyzer (Brain Products, Gilching, Germany). A band pass filter (0.1 and 50 Hz) was applied and the data was down-sampled to 512 Hz. Additionally a 50 Hz notch filter was used to remove 50 Hz noise. For ocular artifact control, we used the independent component analysis (ICA) algorithm infomax (Bell and Sejnowski, 1995; Delorme et al., 2007). By using this algorithm we avoid rejecting large portions of EEG signal due to eye blinks, thus reducing the amount of brain activity subtracted from the measurements (Vigário, 1997; Iriarte et al., 2003). EEG data were then semi-automatically screened for artifacts by the following criteria: maximal voltage step of 50 µV/ms, maximal amplitude of ± 100 µV, values greater than 200 µV per 200 ms interval, activity below 0.5 µV in a 100 ms period. This semiautomatic mode allowed for additional visual inspection and the possibility to reject additional artifacts missed by the software. Artifact free, 15- s long segmented EEG data (see Figure 2) were then exported into MATLAB for further analysis. There were no significant differences between groups in the amount of artifact-free segments included for further analysis (*F*_(2,41)_ = 2.46, *p* = 0.10).

### EEG and statistical analysis: ERS/ERD

Event related spectral perturbation (ERSP) is a measure of a signal’s time-frequency composition changes in a particular frequency range in reference to a baseline time interval. ERSP was computed using the MATLAB toolbox EEGlab (Delorme and Makeig, 2004) using Morlet Wavelets for spectral wavelet transformation. *ERSP*_%_ values were transformed in log-units and converted to decibel unit (dB), by multiplying the log ratio with the factor 10 (Grandchamp and Delorme, 2011). Negative *ERSP*_log_ values which are shown green and red in the plots indicate a decrease in power from baseline which is further called ERD. Positive values plotted in blue indicate increase in power which implies ERS. The Morlet transforms used 3 cycles at the lowest frequencies, 30 at the highest, a time window of 2229 ms and overlap of 27 ms. 300 linear-spaced frequencies from 1.5 Hz to 30 Hz and 400 time points were generated.

The “video-end” trigger marked the end of each video in the EEG recording and was used to extract relevant epochs from the signal. Since each video lasted 7 s, time 0 ms depicts exactly the start of a video. As a baseline interval for the calculation of the ERSP values, a 1-s time window from −2500 ms to −1500 ms before the start of the video was used. The result of these calculations were one ERSP matrix per electrode per participant. Statistical analysis was performed on time-frequency-averaged ERSP values representing larger areas of interest in the frequency range 8–10 Hz from −1000 ms to 7000 in time-intervals of 1000 ms. This was done for all electrodes separately. The 1000 ms before the start of the video marked a baseline period, whereas the first 3 s of the task marked the preparatory phase and seconds four to six marked the motor execution phase (see Figure 2). The 1-s segments were further included in statistical ANOVA analysis as variables. For correlation analysis the mean ERD/ERS values for each phase (baseline vs. preparatory vs. execution) were computed. In order to find outliers within the groups (amateurs, young elite and experts) ERD/ERS values were pooled by group and second in the preparatory phase (second 1 until second 3). ERD/ERS values in three electrode groups (P3, PZ, P4; F3, FZ, F4; C3, CZ, C4) were averaged for each of the three participant group over the preparatory phase resulting in 27 samples. Mean and standard deviation were calculated for each of these samples. Participants who differed more than three standard deviations from the mean were considered to be outliers and excluded from statistical analysis of EEG data. One outlier was found and excluded from the amateurs group.

### Questionnaires

To assess subjective arousal, the SAM questionnaire was used (Bradley and Lang, 1994) as a non-verbal pictorial assessment technique that directly measures arousal along a 9-point scale. Participants rated the videos based on perceived quality of the serve (complexity, rotation, variability and realism) of the opponent. Additionally participants rated the quality and intensity of their own motor imagination. To assess general commitment to the study, the subjects rated motivation, interest in the study, professionalism of the researchers and perceived relevance of the research project. Participants were also asked about the relevance of the forehand top spin within their own playing system and the perceived ability of their own forehand topspin. Rankings by the International Table Tennis Federation served as an objective performance measure for experts^1^.

## Results

### ERD/ERS at the motor cortex

**Figure 3:**
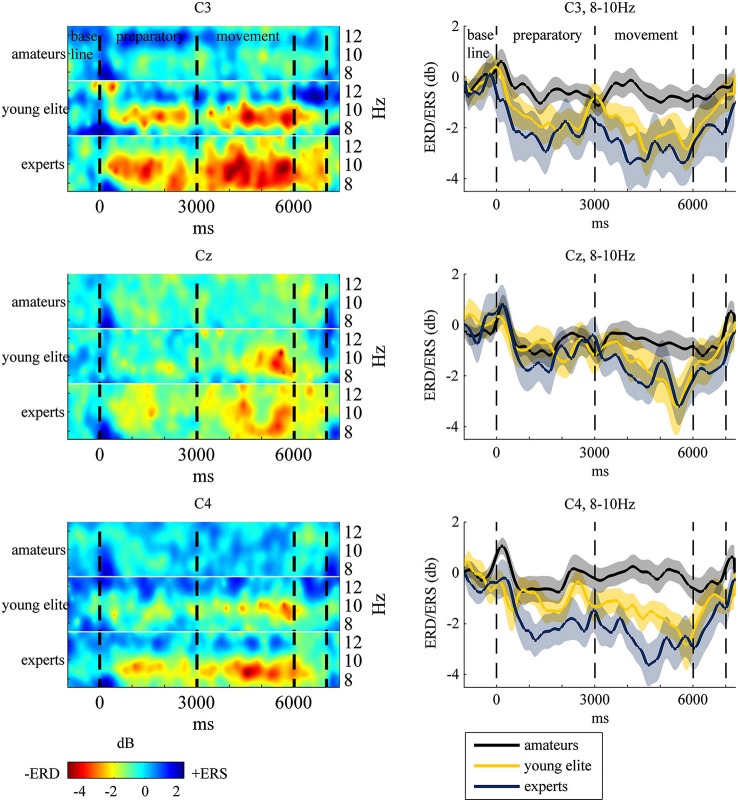
**Left: Event-Related-Spectral-Pertubation (ERSP) plots at central electrodes at 8–12 Hz for each group during the task**. Subjects watched 40 table tennis videos and imagined a specific table tennis stroke. Seconds 1 to 3 comprise the preparatory period and seconds 4 to 6 comprise the movement period. Event-Related-Desynchronization (ERD) and Synchronization (ERS) are indexed by power decrease in red and green (ERD) and power increase in blue (ERS). Right: ERD/ERS in the 8–10 Hz frequency band with standard error of mean (transparent shadow) are displayed over time at the motor cortex for experts (blue line), amateurs (black line) and young elite athletes (yellow line). ERD/ERS is indexed by ERSP values that were transformed to log-units and converted to decibel unit (db). See EEG and statistical analysis: ERS/ERD in Method and Material section for a detailed description.

To test hypothesis (1), that table tennis experts show stronger 8–10 Hz ERD in the motor cortex at the end of the movement period, we analyzed differences in ERD in the motor cortex (C3, CZ, C4) over time between amateurs, young elite athletes and experts. Visual inspection and frequency analysis indicate ERD predominantly in the 8–10 Hz frequency band as expected (see Figure 3). An ANOVA of 8–10 Hz ERD/ERS (dB values) with time (−1000 ms to 6000 ms in 1-s bins), electrode position (C3 vs. Cz vs. C4) and group (experts vs. amateurs vs. young elite athletes) as independent factors showed a significant main effect of time (*F*_(2.89,115.63)_ = 17.50, *p* < 0.001, *partial η*^2^ = 0.30), a significant main effect of group (*F*_(2,40)_ = 4.309, *p* < 0.05, *partial η*^2^ = 0.177) and a significant interaction of time and group (*F*_(5.78,115.63)_ = 2.781, *p* < 0.05, *partial η*^2^ = 0.012)^2^. Bonferroni corrected *post hoc* tests of the main effect of time with aggregated db values over groups and electrode positions showed significant decreases in db values from second -1 (baseline) to second 2 (*p* < 0.001) and second 3 (*p* < 0.01) of preparatory and all other time points of the movement phase (*p* < 0.001). From second 1 of the preparatory phase there were significant decreases to second 2 (*p* < 0.001) of preparation and second 4 (*p* < 0.005), 5 (*p* < 0.001) and 6 (*p* < 0.001) of the movement phase. Further decreases have been shown from the last second of preparation (second 3) to the last second of the movement (second 6, *p* < 0.05) and from first (second 4) to the last second of movement (second 6, *p* < 0.05). The *post hoc* tests of the main effect of group should be regarded with caution because of the significant interaction between time and group. Bonferroni corrected *post hoc* tests with mean db values over all positions and time segments showed significant stronger ERD in experts compared to amateurs (*p* < 0.05) and no differences between experts and young elite athletes (*p* < = 0.69) and between young elite and amateur athletes (*p* = 0.26). We conducted ANOVAs of aggregated db values (mean of db values at C3, Cz, C4) at each time segment to explain the significant interaction of time and group and to assess differences in ERD between groups in the end of the motor execution phase (main hypothesis). When adjusting for multiple comparisons (Bonferroni), only the differences between groups at second 5 of the motor execution phase were significant (*F*_(2,40)_ = 5.94, *p* < 0.01, *partial η*^2^ = 0.23). Further Bonferroni corrected t-tests showed significant stronger ERD in experts compared to amateurs (*p* < 0.01) with a strong effect size (*d* = 1.13), but no differences between experts and young elite athletes (*p* = 0.55) and no differences between young elite athletes and amateurs (*p* = 0.12). Since we a priori stated the strongest differences at the last second of the movement phase (second 6), we also present ANOVA results for this time segment without Bonferroni correction. There was a significant main effect of group (*F*_(2,40)_ = 4.16, *p* < 0.05, *partial η*^2^ = 0.17). Experts showed significantly stronger ERD than amateurs (*p* < 0.05) with a slightly stronger effect size (*d* = 1.17) compared to second 5. There were marginal significant differences between young elite athletes and amateurs (*p* = 0.08) and no differences between experts and young elite athletes (*p* = 0.1).

### ERD/ERS at the fronto-parietal cortex

To test the second part of hypothesis (1) of increased fronto-parietal activation in experts, we conducted an ANOVA of 8–10 Hz ERD/ERS with time (−1000 ms to 6000 ms in 1-s bins), region (frontal vs. parietal), position (F3/P3 vs. Fz/Pz vs. F4/P4) and group (experts vs. amateurs vs. young elite athletes) as independent factors. As at the motor cortex, there was a significant main effect of time (*F*_(2.06,82.72)_ = 23.14, *p* < 0.001, *partial η*^2^ = 0.37), but no significant effects of group (*F*_(1,40)_ = 2.87, *p* = 0.07, *partial η*^2^ = 0.13) and no significant interaction between time and group (*F*_(4.14,82.72)_ = 2.18, *p* = 0.08, *partial η*^2^ = 0.01). But there was a significant interaction between time, region and group (*F*_(8.29,165.88)_ = 3.01, *p* < 0.01, *partial η*^2^ = 0.13). There were no significant interactions between group, time, region and electrode position (left, central, right). Bonferroni corrected *post hoc* tests of the main effect of time with aggregated db values over groups and electrode positions showed significant decreases in db values from second -1 (baseline) to second 1 (*p* < 0.05), second 2 (*p* < 0.001), second 3 (*p* < 0.005) of preparatory and all other time points of the movement phase (*p* < 0.001). From second 1 of the preparatory phase there were significant decreases to second 2 (*p* < 0.001) of preparation and second 4 (*p* < 0.005), 5 (*p* < 0.001) and 6 (*p* < 0.001) of the movement phase. Further decreases have been shown from the last second of preparation (second 3) to seconds 5 (*p* < 0.01) and 6 ( *p* < 0.01) of motor execution and from first (second 4) to the last second of movement (second 6, *p* < 0.05). To test the hypothesis of increased fronto-parietal activation in experts in the end of the motor execution phase, we report the *post hoc* tests of the three-way interaction time x region x group and focus on differences between groups in the last second of the movement phase (second 6). ANOVA at this time point with group (expert vs. amateur vs. young elite) and region (frontal vs. parietal) as independent factors showed no significant main effect of group (*F*_(2,40)_ = 2.90, *p* = 0.07, *partial η*^2^ = 0.13), a significant main effect of region (*F*_(1,40)_ = 30.71, *p* < 0.001, *partial η*^2^ = 0.43) and a significant interaction between region and group (*F*_(2,40)_ = 3.49, *p* < 0.05, *partial η*^2^ = 0.15). The main effect of region indicates significant stronger ERD at the parietal (*M* = −3.43, SD = 3.77) than at the frontal cortex (*M* = −1.65, SD = 2.06). There were no significant differences between groups at the frontal cortex (*F*_(2,40)_ = 2.69, *p* = 0.08, *partial η*^2^ = 0.12) nor at the parietal cortex (*F*_(2,40)_ = 3.07, *p* = 0.06, *partial η*^2^ = 0.13), but there was a significant main effect of group (*F*_(2,40)_ = 3.49, *p* < 0.05, *partial η*^2^ = 0.15) when comparing the contrasts of frontal and parietal ERD (frontal ERD/ERS minus parietal ERD/ERS). Young elite athletes showed significant stronger parietal ERD (relative to frontal) compared to amateurs (*p* < 0.05). There were no significant differences in the parietal ERD (relative to frontal ERD) between experts and amateurs nor between experts and young elite athletes. The results indicate that there is ERD also in the fronto-parietal cortex in experts, amateurs and young elite athletes, but experts did not show significant stronger ERD at frontal or parietal electrodes.

### Activation of the motor cortex and prediction of world rank

**Figure 4:**
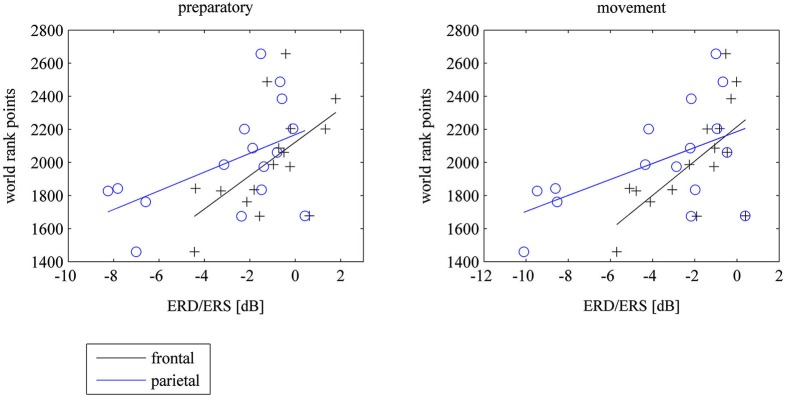
**Scatterplots of mean frontal (F3, Fz, F4) and parietal (P3, Pz, P4) ERD/ERS and world rank points for the preparatory und movement period during the task, where subjects watched 40 table tennis videos and imagined a specific table tennis stroke**. Seconds 1 to 3 comprise the preparatory period and seconds 4 to 6 comprise the movement period. ERD implies power decrease (negative values) and ERS imply power increase (positive values). ERD/ERS is indexed by ERSP values that were transformed to log-units and then converted to decibel unit (db). See EEG and statistical analysis: ERS/ERD in Method and Material section for a detailed description).

There were no correlations between 8–10 Hz ERD at the motor cortex and world rank in experts. However there were significant correlations between 8–10 Hz ERD within the task in frontal and parietal electrodes and world rank points: The weaker the ERD (the stronger the ERS) the more world rank points. The correlations were not different for left, central or right electrode positions. However, they were higher in the preparatory phase at parietal electrodes and higher in the movement phase at frontal electrodes (see Table 1 and Figure 4). There were no correlations in the baseline period.

**Table 1:** **Correlation coefficients between mean frontal (F3, Fz, F4), central (C3, Cz, C4) and parietal (P3, Pz, P4) ERD/ERS and world rank points in experts for baseline (second -1), preparatory (mean second 1 to second 3) and movement (mean second 4 to second 6) period**.

Baseline: *r*(12)			Preparatory: *r*(12)			Movement: *r*(12)		
Frontal	Central	Parietal	Frontal	Central	Parietal	Frontal	Central	Parietal
0.06	−0.10	−0.03	0.60*	0.24	0.53^+^	0.63*	0.40	0.51^++^

*Positive correlation coefficients imply that weaker ERD (or stronger ERS) is correlated with more world rank points in experts*.

*p < 0.05; ^+^p = 0.05; ^++^p < 0.07.

### Control analysis: occipital cortex

To demonstrate, that the stronger ERD in experts compared to amateurs at the motor cortex (hypothesis 1) relies on differences in cerebral motor cortical processes and not on differences in visual attention processes, we conducted an ANOVA of 8–10 Hz ERD/ERS at the occipital cortex at the last second of the movement phase (second 6) with electrode position (O1, O2) and groups (experts, amateurs, young elite athletes) as independent factors. As expected, there were no significant differences between groups (*F*_(2,40)_ = 2.23, *p* = 0.12, *partial η*^2^ = 0.10) and no significant interaction between group and electrode site (*F*_(2,40)_ = 2.06, *p* = 0.14, *partial η*^2^ = 0.09).

### Control analysis: questionnaires

To investigate whether the pressure induction was successful, we conducted an ANOVA to assess differences in subjective arousal (SAM) between groups at the different measurement times. ANOVA indicate a strong main effect of time (*F*_(2,80)_ = 14.747, *p* < 0.001, *partial η*^2^ = 0.27). However neither significant differences between groups nor an interaction of time and skill level emerged, indicating that there were no differences in total arousal between groups nor between groups at the different measurement points. *Post hoc* analyzes indicate a strong increase of arousal after the pressure induction (*p* < 0.005) but no significant increase in arousal after the first 20 video trials (*p* = 0.12), verifying, that all participants experienced an increase in performance pressure. There were no significant differences between groups in the perception of the expertise of the player in the videos (*F*_(2,40)_ = 1.83, *p* = 0.17, *partial η*^2^ = 0.08). There were no differences between groups in the perceived quality of the serve (*F*_(2,40)_ = 0.464, *p* = 0.63, *partial η*^2^ = 0.02). Overall, amateurs (*M* = 2.79, SD = 0.82) rated their imagination quality slightly more vivid than experts (*M* = 2.45, SD = 0.81) and young elite athletes (*M* = 2.2, SD = 0.79), but these differences were not statistically significant (*F*_(2,40)_ = 0.13, *p* = 0.18, *partial η*^2^ = 0.08). Young elite athletes showed the highest commitment (*M* = 3.3, SD = 0.51), similar to amateurs (*M* = 3.29, SD = 0.31) and higher than experts (*M* = 2.87, SD = 0.58). These differences between groups were significant (*F*_(2,40)_ = 3.72, *p* < 0.05, *partial η*^2^ = 0.16), however bonferroni corrected *post hoc* tests showed no significant differences between experts and amateurs (*p* = 0.08), between amateurs and young elite athletes (*p* = 0.1) and only marginal significant differences between experts and young elite athletes (*p* = 0.06). Neither the relevance of the forehand topspin (*F*_(2,40)_ = 1.32, *p* = 0.29, *partial η*^2^ = 0.06) nor the perceived expertise of the forehand topspin differed significantly between groups (*F*_(2,40)_ = 0.45, *p* = 0.64, *partial η*^2^ = 0.02). We further assessed, if the commitment to the study, arousal during the task and the imagination quality is correlated with ERD in the motor and fronto-parietal cortex. We correlated mean motor and mean fronto-parietal ERD during the preparatory phase and the movement phase with these three control variables. Only the commitment to the study showed small positive correlation coefficients with motor ERD, indicating a slight association between higher commitment to the study and weaker ERD at the motor cortex. However, after controlling for multiple comparisons (Bonferroni), these correlations did not reach significance and were thus not included as a covariates into the main analysis.

## Discussion

We tested the hypothesis that expert compared to amateur table tennis athletes exhibit stronger activation (indexed by stronger 8–10 Hz alpha ERD) in the motor and fronto-parietal cortex during a motor imagery task, in which athletes had to react to a table tennis stroke presented in different video clips. We expected an intermediate activation level in a third group with an intermediate skill level (young elite athletes). To our knowledge to this date there is no study assessing differences in ERD between groups of different skill levels in high reactive sports like table tennis. Since elaborated attentional skills and multisensory integration are important features of motor skills in table tennis, we argued that there are differences in motor and fronto-parietal cortex activation. We further assumed ERD in the low-frequency 8–10 Hz band reflecting motor and motor attentional processes during complex motor tasks. To demonstrate that ERD in the motor cortex predicts performance in elite athletes, we assumed a positive correlation between the activation of the motor cortex (stronger ERD) and world rank points.

The results demonstrate a significantly stronger 8–10 Hz ERD in experts compared to amateurs in the motor cortex (C3, Cz, C4) at the end of the motor execution phase with an intermediate 8–10 Hz ERD in young elite athletes. There was a trend towards stronger 8–10 Hz ERD in the frontal and parietal cortex in experts and young elite athletes compared to amateurs, however these differences were not significant. Contrary to our expectation, less activation or even cortical inhibition in the fronto-parietal cortex was correlated with more world rank points during motor preparation and execution. This association was task-dependent, since there was no correlation between 8–10 Hz ERD in the baseline period and world rank points.

The data suggests that 8–10 Hz ERD in the motor cortex might reflect a physiological mechanism of motor skill in table tennis. SMR and alpha rhythms are generated by thalamo-cortical and cortico-cortical loops that control the access and retrieval of stored sensorimotor and cognitive information (Pfurtscheller and Lopes Da Silva, 1999) and the functional meaning of low frequency 8–10 Hz ERD seems to be related to general motor attention and readiness during motor tasks (Klimesch, 1999; Pfurtscheller and Lopes Da Silva, 1999; Pfurtscheller et al., 2000; Klimesch et al., 2007). The present results indicate that high motor skills in table tennis imply selective engagement of thalamocortical and cortico-cortical loops for motor-attentional performance: excitability of the motor cortex during motor reaction, planning and execution with high attentional demands and, once elite expertise is reached, less activation of the fronto-parietal attention network. Motor cortical excitability indicates higher focal neuronal firing, which is often observed after motor learning (Koeneke et al., 2006b; Cirillo et al., 2011).

Our finding of a stronger activation of the motor cortex contradicts several studies showing that elite athletes (shooters, karateka, gymnasts, kendoists) require “less” brain activation (weaker ERD) in task relevant brain areas compared to novices during sport specific tasks (Kita et al., 2001; Di Russo et al., 2005; Del Percio et al., 2008, 2009a; Babiloni et al., 2009). These authors state a more efficient cortical function in more skilled individuals by means of less cortical activation (weaker ERD) when skills are highly elaborated and become automatic (“neural efficiency hypothesis”). These assumptions are also based on studies showing a reduction of brain activity during cognitive tasks with the development of expertise (Grabner et al., 2006; Dunst et al., 2014) and on motor skill learning tasks showing a reduction in brain activity in the motor cortex from pre- to posttraining measurements indicating an optimization of cortical resources (Haufler et al., 2000; Jäncke et al., 2006; Koeneke et al., 2006a). The association of less fronto-parietal activation (weaker ERD) with higher world rank points in elite experts in our study can be interpreted in terms of the “neural efficiency hypothesis”: the better the performance of elite table tennis players the fewer attentional resources they require for motor tasks with high attentional demands. Less attentional resources and executive control processes are often observed also in slow motor learning tasks (for a review see Dayan and Cohen, 2011).

The stronger activation of the motor cortex in elite experts compared to amateurs in our study resembles an EEG study with karate and fencing athletes that found stronger ERD in elite compared to novice athletes during a sport specific visual integration task (Del Percio et al., 2007) and functional imaging studies with musicians that showed stronger activations in the contralateral primary sensory cortex in expert violinists compared to amateurs (Lotze et al., 2003). Further, our results are in line with studies looking at cortical activation patterns of complex motor learning tasks (Karni et al., 1995, 1998; Hazeltine et al., 1997; Rauch et al., 1997; Seidler et al., 2005) and complex motor visual multisensory integration tasks (Waldschmidt and Ashby, 2011), that showed increased cortical activity in task-relevant areas, when expertise or automaticity was developed (for reviews see Dayan and Cohen, 2011; Chang, 2014). Several motor learning studies using fMRI or PET showed an initial decrease of activity at the primary motor region (M1) in the first motor learning stage, further an increase in activation of M1, supplementary motor area (SMA), whole motor cortex and other subcortical areas after 3–5 weeks of training when motor skills become implicit and automatic (Karni et al., 1995; Inoue et al., 2000; Floyer-Lea and Matthews, 2004, 2005; Xiong et al., 2009). Contradictory evidence also exists (Hlustík et al., 2004; Pollok et al., 2014). A recent experimental motor learning study in monkeys showed reduced metabolic activity at M1 after training only when the motor task was fully self-generated and not visually guided (Picard et al., 2013). We argue, that table tennis is highly similar to complex motor tasks that require perception, visual attention and multisensory integration. Therefore it differs from the sport disciplines such as rifle shooting, golf and gymnastics, which have been the focus of investigations using EEG and analyzing ERD/ERS during motor actions in elite experts. These sport disciplines are rather characterized by simple, self-generated movements with low attentional demands. We argue, that the stronger and focused activation of the motor cortex in table tennis athletes found in our study is a physiological mechanism in highly developed motor skills in complex motor tasks with high attentional demands that require anticipation, multi-sensory integration and fast reactions.

Further, our study extends recent neuroimaging findings on motor expertise in action observation (for a review see Turella et al., 2013). There is strong evidence that elite experts of several disciplines like archery (Kim et al., 2011), badminton (Wright et al., 2010) or basketball (Abreu et al., 2012) showed stronger activation in brain regions typically involved in action observation during observation of sport specific movements. In two FMRI studies (Calvo-Merino et al., 2005, 2006), Calvo-Merino et al. (2005) could demonstrate that ballet dancers and capoeira fighters showed stronger activation for the observation of trained in comparison to untrained dance styles in brain regions (bilateral premotor cortex, bilateral superior parietal lobule, and anterior intraparietal sulcus, left ventral premotor cortex and left superior temporal sulcus) typically involved in action observation (Rizzolatti and Craighero, 2004; Caspers et al., 2010; Rizzolatti and Sinigaglia, 2010). There were no such differences in non-dancers. Calvo-Merino et al. (2006) further showed, that activity within the left premotor cortex and the bilateral anterior intraparietal sulcus was higher when observing actions within the observer’s motor repertoire emphasizing an effect of motor expertise rather than visual familiarity with the dance styles presented. The result of a stronger engagement of the motor cortex during action observation and motor imagery in our study is thus in line with the results of a stronger engagement of task relevant brain areas in highly-skilled athletes or dancers during action observation of skilled and familiar motor actions in their discipline.

### Limitations

It should be noted, that due to the constraints of EEG (spatial resolution) and the small number of electrodes, we cannot exactly conclude that the source of the 8–10 Hz ERD is the motor cortex (SMR). Nevertheless, the differences between experts and amateurs were only significant in the motor cortex with no differences between groups at the occipital cortex. Consequently, we argue that the source of the ERD is the cerebral motor system. Also, the EEG data does not allow us to analyze localized functional topographical details in motor or parietal cortex compared. However, we decided to use EEG in order to get exquisite temporal resolution needed for the specific task demands of table tennis which comprises short reaction times and fast movements. We also aimed to extend the existing literature of ERD/ERS in elite athletes to indicate that ERD serves as a physiological mechanism of elaborated motor skills in table tennis. Another apparent limitation is the difference in age between young elite athletes, experts and amateurs. There is some evidence that movement related ERD in young children (around 7 years of age) is weaker compared to adults during motor tasks (Pangelinan et al., 2011). Thus we included only children that most probably already reached puberty (14 years of age and older). However, we still cannot rule out the possibility, that the age of the young elite athletes might influenced our results. Another limitation is the small sample size (*N* = 14) especially for the correlation analysis. Thus also these analysis should be regarded as preliminary which needs replication. These results should therefore be interpreted with caution. However, the strong effect sizes of the differences between experts and amateurs at the motor cortex and the high correlation coefficients underpin the quality of the results.

## Conclusions

In conclusion, the present study shows that 8–10 Hz ERD is stronger in elite table tennis players compared to amateurs at the motor cortex and a weaker 8–10 Hz ERD in the fronto-parietal cortex is associated with more world rank points in experts. These results suggest that high motor skills in table tennis are associated with focused excitability of the motor cortex during reaction, movements planning and execution with high attentional demands. Among elite experts however, less activation of the fronto-parietal attention network may be necessary to become a world champion.

## Author and contributors

We declare that all authors substantially contributed to the conception or design of the work, or the acquisition, analysis, or interpretation of data for the work and drafting the work or revising it critically for important intellectual content. All authors further approved the version to be published and agreed to be accountable for all aspects of the work in ensuring that questions related to the accuracy or integrity of any part of the work are appropriately investigated and resolved. More specifically, authors contributed with the following focuses:

Sebastian Wolf: Design of the work, application for funding, data acquisition, data processing, data analysis, drafting the work, revision.

Ellen Broelz: data acquisition (amateurs, young elite athletes), interpretation of the work, drafting.

David Scholz: data acquisition, data processing, data analysis, drafting (figures) and ERD/ERS.

Ander Ramos Murguialday: conceptualizaion, data processing (MATLAB Code), data analysis, drafting, revision.

Philipp M. Keune: conceptualization, data analysis, drafting.

Martin Hautzinger: design of the work, data interpretation, drafting, revision.

Niels Birbaumer: design of the work, data interpretation, drafting, revision.

Ute Strehl: design of the work, data interpretation, drafting, revision.

## Conflict of interest statement

The authors declare that the research was conducted in the absence of any commercial or financial relationships that could be construed as a potential conflict of interest.

## Footnotes

^1^Reliable and valid performance indices for young elite and amateur players cannot be provided because there are no consistent ranking systems for these athletes.

^2^All results have been corrected for violations of sphericity (Greenhouse-Geisser).
